# Fish consumption and the risk of gastric cancer: systematic review and meta-analysis

**DOI:** 10.1186/1471-2407-11-26

**Published:** 2011-01-20

**Authors:** Shengjun Wu, Jie Liang, Lei Zhang, Xia Zhu, Xufeng Liu, Danmin Miao

**Affiliations:** 1 Department of Psychology, School of Aerospace Medicine, Fourth Military Medical University, Xi'an, Shaanxi, PR China; 2 State Key Laboratory of Cancer Biology & Xijing Hospital of Digestive Diseases, Fourth Military Medical University, Xi'an, Shaanxi, PR China; 3 Department of Epidemiolody, School of Military Preventive Medicine, Fourth Military Medical University, Xi'an, Shaanxi, PR China

## Abstract

**Background:**

Gastric cancer is the fourth most frequently occurring malignancy after lung, breast, and colorectal cancer, and the second most common cause of death from cancer worldwide. Epidemiologic studies have examined the possible association between fish consumption and gastric cancer, but the results were inconclusive. We conducted a systematic review and meta-analysis to examine the association between fish intake and the risk of gastric cancer.

**Methods:**

PubMed was searched for studies published in English-language journals from 1991 through 2009. We identified 17 epidemiologic studies (15 case-control and 2 cohort studies) that included relative risks (RRs) or odds ratios (ORs) estimates with 95% confidence intervals (CIs) of the relationship between gastric cancer and fish consumption. Data were extracted using standardized data forms. Summary RRs or ORs for the highest versus non/lowest fish consumption levels were calculated using random-effects model. Heterogeneity among studies was examined using Q and I^2 ^statistics.

**Results:**

In this study, 5,323 cases of gastric cancer and over 130,000 non-cases were included. The combined results from all studies indicated that the association between high fish consumption and reduced gastric cancer risk was not statistically insignificant (RR = 0.87, 95% CI = 0.71-1.07).

**Conclusions:**

Current evidence indicated that the association between fish consumption and risk of gastric cancer remains unclear.

## Background

Gastric cancer is the fourth most frequently occurring malignancy, after lung, breast and colorectal cancer, and the second most common cause of death from cancer worldwide [1,2]. It has been estimated that 1 million patients are newly diagnosed with gastric cancer worldwide each year, with 700,000 patients dying from this disease annually [1].

Diet has been found to be important factor in the development of gastric cancer [3-6]. A report published in 2007 by the World Cancer Research Fund and the American Institute for Cancer Research on the relationship between diet and cancer suggested that the consumption of certain types of food may be directly associated with the development of this disease [7].

Fish is part of the usual diet of most people worldwide and is an ideal source of n-3 fatty acids, which are important components of cell membranes [8]. These long-chain fatty acids have been reported to suppress mutations, inhibit cell growth, and enhance cell apoptosis, thus reducing the risk of cancer [9-11]. To date, however, there have been no intervention studies examining the association between fish consumption and the risk of gastric cancer. Although several epidemiological studies have focused on this association, their conclusions have been inconsistent [12-28]. We therefore preformed a systematic review and meta-analysis to assess the association between fish consumption and gastric cancer risk.

## Methods

### Data Sources and Searches

We comprehensively searched through PubMed for all medical literature published in English-language journals up to January 2009. In searching outcomes, we used the search terms stomach neoplasms[MeSH] OR gastric neoplasms[tiab] OR stomach cancer[tiab] OR gastric cancer[tiab] OR stomach carcinoma[tiab] OR gastric carcinoma[tiab] OR stomach tumor[tiab] OR gastric tumor[tiab]. In searching exposure, we used the terms food[MeSH] OR diet[MeSH] OR diets[tiab] OR dietetic[tiab] OR dietary[tiab] OR eating[tiab] OR intake[tiab] OR nutrient[tiab] OR nutrition[tiab] OR fish[tiab] OR seafood[tiab]. For the search on study design, we used the terms case-control studies [MeSH] OR retrospective studies [MeSH] OR cohort studies [MeSH] OR prospective studies [MeSH]. In addition, we reviewed the reference lists from all relevant articles to identify additional studies.

### Study Selection

Studies that met the following criteria were included in the meta-analysis: 1) the study was either a case-control or cohort study; 2) the exposure of interest was fresh fish consumption; 3) the study reported the number of gastric cancer cases and controls or non-cases; and 4) the study reported relative risks (RRs) or odds ratios (ORs) with their corresponding 95% confidence intervals (CIs) for the highest versus non/lowest level of fish intake. Two authors (Zhu and Liu) screened all references; if they differed as to whether to include a study, that study was discussed by all authors, with decision to include based on voting.

### Data Extraction and Quality Assessment

If data were duplicated in more than 1 study, the most recent study was included in the analysis. We identified 18 potentially relevant articles concerning fish consumption and gastric cancer risk [12-29]. Three authors (Wu, Liang and Zhu) performed data extraction and quality assessment; again any discrepancies were settled by a discussion and vote by all authors.

One study was excluded because fish consumption was compared between meat eaters and non-meat eaters [29]. Seventeen studies were included in the meta-analysis of fish intake and gastric cancer: 15 case-control studies and 2 cohort studies. We used a standardized protocol and reporting form to abstract the following data from each publication: reference (first author, year of publication), study design, country in which the study was performed, numbers of cases and non-cases, lowest and highest levels of fish consumption, the RR or OR with 95% CI for gastric cancer associated with fish intake, covariate adjustments, the methods used for dietary assessment.

### Data Synthesis and Analysis

Study-specific ORs/RRs with corresponding 95% CIs for highest versus non/lowest fish consumption levels were extracted. If the study provided both different and general estimates, we included only the general results. For example, if the study included ORs/RRs and 95% CIs of males and females and both genders combined, we included only the combined data in our meta-analysis. For case-control studies, the proportions (expressed as percentages) of control subjects in the highest and non/lowest consumption categories were included. For cohort studies, the percentages of subjects in categories of the highest and non/lowest consumption levels were calculated as the number of subjects in each of these 2 categories relative to the total number of study subjects.

Q and Higgins I^2 ^statistics were used to examine heterogeneity not only among studies but also between the subgroups included in this meta-analysis [30,31]. For the Q statistics, P < 0.10 indicated statistically significant heterogeneity [30]. We defined statistical significance as P < 0.10 rather than the conventional level of 0.05 because of the low power of this test [32]. I^2 ^values lie between 0% (no observed heterogeneity) and 100% (maximal heterogeneity); thus, an I^2 ^value greater than 50% may be considered to represent substantial heterogeneity [31]. Risk estimates were calculated using a random-effects model, incorporating both within- and between-study variability [30]. Summary estimates were calculated for each type of study design (case-control and cohort) as well as for their combination.

To assess the potential for publication bias, we used funnel plots and Egger's regression [33,34]. All statistical analyses were performed with Stata (Version 10.0; StataCorp, College Station, TX).

## Results

### Literature Search

**Figure 1:**
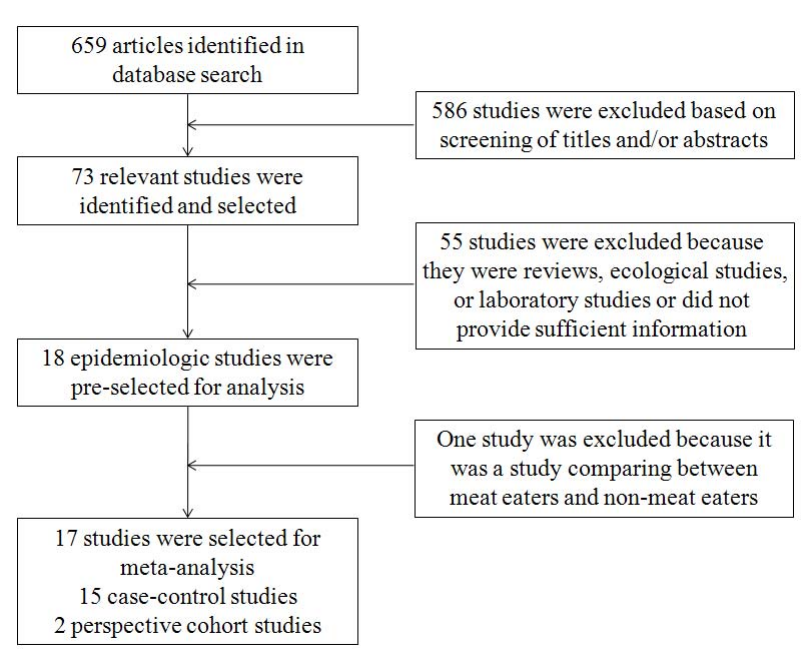
**Flowchart of Selection of Studies for Inclusion in Meta-analysis**.

The detailed steps of our literature search are shown in Figure 1. In brief, our search found that, of 659 potential articles, 73 concerned fish consumption and the risk of gastric cancer. Of these 73, 55 were excluded because they were review articles, ecological or laboratory studies, or did not provide sufficient information. One study was excluded because it compared meat eaters and non-meat eaters [29]. We also searched all of references in the 73 relevant articles for additional studies. Finally, 17 studies were included in the meta-analysis.

### Study Characteristics

The 17 articles that met the inclusion criteria for our meta-analysis were published between 1991 and 2009 and involved a total of 5,323 cases and 130,903 non-cases. Of these 17 studies, 8 were population-based case-control studies [12,13,18-20,22,25,26], 7 were hospital-based case-control studies [14-17,21,23,24], and 2 were cohort studies [27,28].

### Meta-Analysis

**Figure 2:**
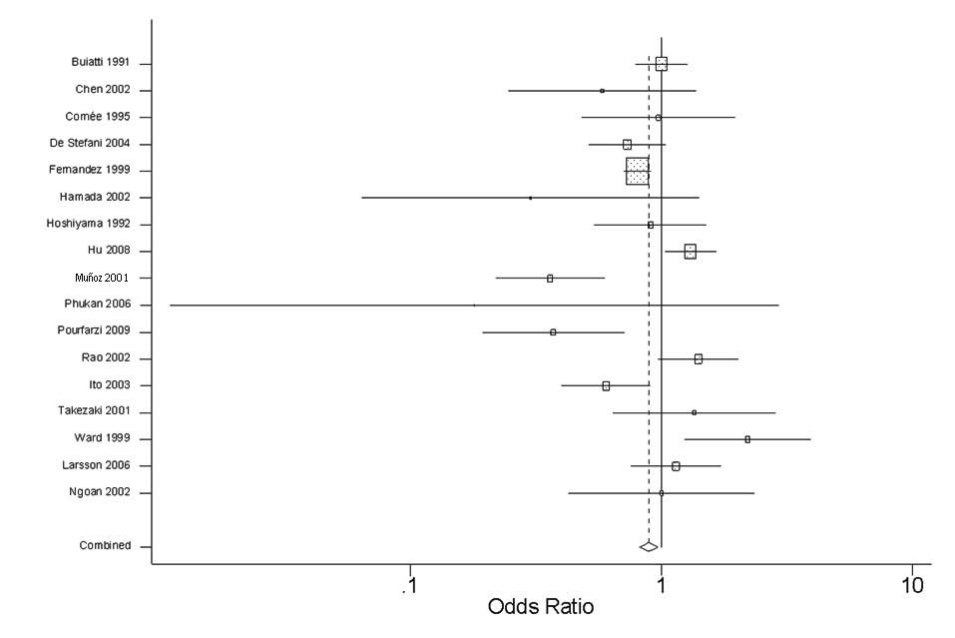
**Meta-analysis of All Studies of Gastric Cancer Risk**.

The lowest and highest levels of fish consumption and the RR or OR for each of the 17 included studies, along with their summary OR, are shown in Table 1 and Figure 2.

**Table 1:** Characteristics of Studies Included in the Meta-analysis

Reference	Design	Country	No. of Cases/No. of Non-cases	OR or RR (95% CI)	Fish Consumption Levels	Covariate Adjustment	Methods Used for Assessing Dietary Dntake
Buiatti 1991	PCC	Italy	510/1159	1.00 (0.80-1.30)	T3 vs. T1	Age, sex, area, place of residence, migration, socioeconomic status, familiar GC history, Quetelet index, total caloric intake	Interview with a structured questionnaire
Chen 2002	PCC	China	124/449	0.58 (0.25-1.40)	Q4 vs. Q1	Age, sex, energy intake, respondent type, BMI, alcohol use, tobacco use, eduction, family history, vitamin supplement use	Interview with a modified version of the short Health Habits and History Questionnaire
Cornée 1995	HCC	France	92/128	0.97 (0.48-1.96)	Q3 vs. Q1	Age, sex, occupation and total energy intake	Interview with a dietary history questionnaire
De Stefani 2004	HCC	Uruguay	240/960	0.73 (0.51-1.03)	T3 vs. T1	Age, sex, residence, urban/rural status, education, boby mass index, toal energy intake	Interview with a food frequency questionnaire (FFQ)
Fernandez 1999	HCC	Italy	745/7990	0.80 (0.70-0.90)	Increment 1 serving/wk vs. 1 Serving/wk	Age, sex, area of residence, education, smoking, alcohol consumption, body mass index	Interview with a structured questionnaire
Hamada 2002	HCC	Brazil	96/192	0.30 (0.10-2.20)	Daily vs. <1 d/week	Beef comsuption, country of birth	Interview with a questionnaire
Hoshiyama 1992	PCC	Japan	216/483	0.90 (0.50-1.40)	≥15/week vs. ≤4/week		Interview with a lifestyle questionnaire
Hu 2008	PCC	Canada	1182/5039	1.3 (1.00-1.60)	Q3vs. Q1 (≥5 oz/week vs. ≤2 oz/week)	Age, province, education, body mass index, sex, alcohol use, pack-year smoking, total of vegetable and fruit intake	Mail with a short version of the Block food frequency questionnaire (FFQ)
Muñoz 2001	PCC	France	302/485	0.36 (0.22-0.60)	Q4 vs. Q1	age, sex, tobacco, alcohol, total calories and SES	Interview with a semi-quantitative food frequency questionnaire (FFQ)
Phukan 2006	HCC	India	329/665	0.18 (0.02-5.30)	≥2/week vs. never	Level of education, tobacco use, alcohol drinking,	Interview with a structured pretested questionnaire
Pourfarzi 2009	PCC	Iran	210/389	0.37 (0.19-0.70)	≥1/week vs. never or infrequently	Gender, age group, education, family history of GC, citrus fruit, garlic, onion, red meat, fish, diary products, strength and warmth of tea, preference for salt intake and H. pylori	Interviewed with a structured questionnaire
Rao 2002	HCC	India	119/1591	1.4 (0.95-2.00)	At least once a week vs. never or once in 2 weeks	Habit, age group and sex	Interview with a questionnaire
Ito 2003	HCC	Japan	508/36490	0.60 (0.40-0.90)	≥ 5 times/week vs. < 1 time/week	Age, year, season at first hospital visit, smoking habit and family history of gastric cancer	Interview with a self-administered questionnaire
Takezaki 2001	PCC	China	187/333	1.35 (0.64-2.85)	≥3 times/week vs. <1 time/month	Age, sex, smoking and drinking	Interview with a structured questionnaire
Ward 1999	PCC	Mexico	220/752	2.20 (1.20-3.80)	≥2.6 times/week vs. <1 time/week	Age, gender, total calories, chilli pepper consumption, added salt, history of peptic ulcer, cigarette smoking and socioeconomic status	Interview with a semiquantitative food frequency questionnaire
Larsson 2006	cohort	Sweden	136/61433	1.14 (0.75-1.72)	Highest vs. lowest	Age, education, body mass index, intake of total energy, alcohol, fruits and vegetables	Mail with a food-frequency questionnaire (FFQ)
Ngoan 2002	cohort	Japan	107/12365	0.90 (0.30-2.10)	≥1 time/day vs. ≤2-4 time/month	Sex, age, smoking and other dietary factors	Interview with a self-administered questionnaire

Abbreviations: PCC: population-based case-control study; HCC: hospital-based case-control study; Q: quartile; T: tertile; OR: Odd Risk, RR: Relative Risk; CI: Confidence Interval

We observed significant heterogeneity in the results of these 17 studies (Q = 59.92, P < 0.001, I^2 ^= 73.3%). The summary OR for all of the studies showed that high fish consumption was not associated with a reduction in risk of gastric cancer (summary RR = 0.87, 95% CI = 0.71-1.07).

Significant heterogeneity was found among the 15 case-control studies (Q = 58.39, P < 0.001, I^2 ^= 76.0%) but not between the 2 cohort studies (Q = 0.07, P = 0.79, I^2 ^= 0.0%). Similar to the results from all studies combined, there was no significant association between fish intake and gastric cancer risk either in the case-control (summary OR = 0.85, 95% CI = 0.68-1.06) or cohort (summary OR = 1.11, 95% CI = 0.77-1.62) studies (Table 2).

**Table 2:** Meta-analysis of Fish Consumption and Gastric Cancer Risk

Category of Studies	No. of Studies	Summary OR or RR (95% CI)	**I**^**2**^
All studies	17	0.87 (0.71-1.07)	73.3%
Case-control studies	15	0.85 (0.68-1.06)	76.0%
Population-based case-control studies	7	0.87 (0.60-1.27)	82.1%
Hospital-based case-control studies	8	0.82 (0.63-1.05)	56.3%
Population-based versus hospital-based case-control studies			0.0%
Cohort studies	2	1.11(0.77-1.62)	0.0%
Case-control versus cohort studies			31.4%
Western studies	9	0.92 (0.71-1.19)	80.1%
Eastern studies	8	0.80 (0.54-1.16)	64.3%
Western versus Eastern studies Mail assessment Interview versus mail assessment			0.0%
Interview assessment	15	0.82 (0.66-1.02)	69.7%
Mail assessment	2	1.26 (1.03-1.55)	0.0%
Interview versus mail assessment			73.3%

Abbreviations: OR: Odd Risk, RR: Relative Risk; CI: Confidence Interval

When we stratified the various studies by design (case-control versus cohort), we found no significant heterogeneity between the 2 types of study design (Q = 1.46, P = 0.23, I^2 ^= 31.4%), or between population-based and hospital-based case-control studies (Q = 0.07, P = 0.80, I^2 ^= 0.0%). However, significant heterogeneity was found among both the population-based (Q = 39.16, P < 0.001, I^2 ^= 82.1%) and hospital-based (Q = 13.73, P = 0.033, I^2 ^= 56.3%) case-control studies.

When we stratified the studies geographically (Western versus Asian countries), we also found no significant heterogeneity (Q = 0.35, P = 0.55, I^2 ^= 0.0%). However, significant heterogeneity was found among studies performed in Western (Q = 40.14, P < 0.001, I^2 ^= 80.1%) and Asian (Q = 19.59, P = 0.007, I^2 ^= 64.3%) countries. We found no association between fish intake and gastric cancer risk in either Western (summary OR = 0.92, 95% CI = 0.71-1.19) or Asian (summary OR = 0.80, 95% CI = 0.54-1.16) countries. When the studies were stratified by method of dietary assessment (face-to-face interview with a questionnaire versus mailed questionnaire), there was significant heterogeneity between the two methods (Q = 7.95, P = 0.01, I^2 ^= 87.4%). Significant heterogeneity was also found among studies using interview assessments (Q = 46.26, P = 0.00, I^2 ^= 69.7%) but not among those using mailed questionnaires (Q = 0.29, P = 0.59, I^2 ^= 0.0%). Results of the studies using interviews showed no significant association between fish intake and gastric cancer (summary OR = 0.86, 95% CI = 0.66-1.02). However, fish consumption was a risk factor for gastric cancer in studies using mailed questionnaires (summary OR = 1.26, 95% CI = 1.03-1.55) (Table 2).

### Publication Bias Analysis

**Figure 3:**
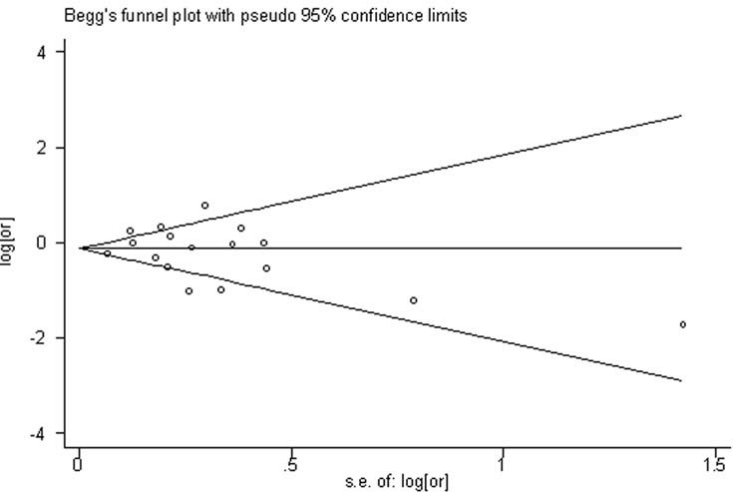
**Begg's Funnel Plot of Fish Consumption and Risk of Gastric Cancer**.

Figure 3 shows a symmetric Begg's funnel plot, indicating that there was no publication bias (P = 0.51). Further, there was no evidence of bias using Egger's test (intercept = -0.08, P = 0.59).

In addition to the studies used in this meta-analysis, other studies provided potentially important information about fish consumption and gastric cancer in formats other than RR or OR with 95% CI [35-37]. Those results are shown in Table 3.

**Table 3:** Other Studies Including Information of the Association between Fish Consumption and Risk of Gastric Cancer

Study	Design	Methods	Country	No. of Cases/No. of Non-cases	Information Provided
Campbell, 2008	PCC	Factor analysis	Canada	1169/2332	The loading score of fresh fish is larger than 25 which means fish is a protective factor for gastric cancer
Nomura, 2003	PCC	Geometric mean comparison	USA	230/446	No remarkable differences in the intake of fish between case and control group
Kim, 2004	Cohort	Factor analysis	Japan	400/41712	Fish consumption loads more on traditional dietary pattern than healthy dietary pattern in male and female respectively. healthy pattern decreased the risk of gastric cancer among females, while the traditional pattern increased the risk in both genders

Abbreviations: PCC: population-based case-control study; HCC: hospital-based case-control study

## Discussion

The important role played by diet in preventing cancer has received much attention in recent years [38-44]. Our meta-analysis evaluated the strength of current evidence for the effect of fish consumption on the lowering of gastric cancer risk, using studies published in recent years. Most of these studies were primarily designed to investigate the effect of fish consumption on gastric cancer risk. Our meta-analysis was strengthened by the detailed information provided in our study design, including the selection criteria for cases and controls and the methods of data collection.

Overall, the summary OR for all of the studies showed no significant association between fish consumption and the risk of gastric cancer, and no such association was found when the case-control or cohort studies were evaluated individually. There was a statistically significant heterogeneity among the 17 studies on fish consumption and gastric cancer risk. In analyzing risk estimates and 95% CI, we only used a random-effects model that considered both intra- and inter-study variation. This random-effects model is more conservative, and hence more appropriate, than a fixed-effects model. Thus, our results were derived from the random-effects model, regardless of their condition of homogeneity. Indeed, we found that, across homogeneous studies, the fixed-effects and random-effects models provided similar results.

Using the funnel plots and Egger's method, we observed no publication bias of the effects of fish consumption on risk of gastric cancer.

One of the 17 studies provided data on consumption of fresh fish and other types of processed fish, but we selected only data on fresh fish consumption [22]. Although there is no conclusive evidence on the association between processed fish intake and risk of gastric cancer, many epidemiological studies and reviews have found that consumption of highly salted food was strongly associated with the risk of gastric cancer [45-51]. This may be due to the presence in highly salted foods, such as salted or smoked fish or processed foods, of chemical carcinogens such as nitrites and their related compounds, or of heterocyclic amines, which have been detected in fish or meat cooked at high temperatures, such as grilling [52-58]. In addition to salt and nitrites, 2-chloro-4-methylthiobutanoic acid, a mutagen present in salted fish, may be associated with gastric carcinogenesis [59].

One study reported the OR and 95% CI of intestinal and other types of gastric cancer; we selected only the data for intestinal gastric cancer [12]. Gastric cancer can be divided into two histological classifications, intestinal and diffuse, which differ in histology, epidemiology, genetic profile, and clinic outcome [60]. The relative frequencies of intestinal and diffuse type cancer have been found to range from 54-72% and from 10-31%, respectively [61]. More important, many studies have found that the intestinal-type is linked more closely to dietary and environmental risk factors [62-64].

From one study [28], we selected the RR with 95% CI after excluding patients followed-up less than three years because the preclinical symptoms of stomach cancer might have caused a change in diet and thereby biased the results [65,66]. In another study [21], which provided both univariate and multivariate OR with 95% CI, we selected the data derived from multivariate analysis because univariate OR and 95% CI were obtained by conditional logistic regression analysis, whereas multivariate OR and 95% CI were obtained after controlling for many additional factors, including level of education and tobacco use.

Animal model studies have shown that fatty acids can affect the risk of developing cancer [67,68]. Fish and fish oil are rich sources of n-3 fatty acids and may have anti-inflammatory potential inhibiting the growth of lung, breast, and colon cancer [69-73]. There may be reasons for the discrepancies observed between our results and the findings of other studies. For example, consumption of freshwater but not marine fish was found to be related to an increased risk of breast cancer [74]. Freshwater fish contain lower levels of omega-3 but higher levels of omega-6 fatty acids than marine fish, and omega-6 fatty acids were found to have no significant association with breast or colorectal cancer [75-77]. Most of the studies included in our meta-analysis, however, did not specify what type of fish was consumed.

Second, different methods were used for dietary assessment. Of the 17 studies we included, 4 used the food frequency questionnaire (FFQ) to assess dietary intake [15,19,20,27]. This questionnaire has an important role in chronic disease epidemiology and has become the dominant method for assessing food consumption in epidemiological studies [78]. A multi-cultural epidemiologic study has indicated that this questionnaire has reasonable reliability and validity [79]. Two other studies used other dietary questionnaires that have been found to have good reliability and validity [16,26]. The other included studies, however, did not provide enough information about the questionnaires they used [12-14,17,18,21-25,28]. In addition, 2 studies used mailed questionnaires to assess dietary patterns, while the other 15 used interview assessments. The results of the 2 studies using mailed questionnaires indicated that fish consumption increased gastric cancer risk, although underlying factors, such as response rate to mailed questionnaires, may have affected these results.

The third reason may be our inclusion of relatively few studies in our meta-analysis. Additional epidemiological studies are needed to obtain definitive results concerning the association between fish intake and gastric cancer risk.

Although our search identified additional studies with useful information on the association between fish intake and gastric cancer risk, these studies were not included in this analysis. For example, factor analysis showed that the loading score of fresh fish was >25, indicating that fish intake was a protective factor against gastric cancer [35]. However, this type of data could not be combined with the other data in our meta-analysis. The exclusion of these useful studies may have influenced our results. We found that the summary RR was 0.87 (95% CI = 0.71-1.07), suggesting that fish consumption has a marginal preventive effect on the risk of gastric cancer. Inclusion of those other studies may have altered our results.

Similar to all meta-analyses, our meta-analysis has limitations resulting from the availability, quality, and heterogeneity of the published data, and these limitations should be considered when interpreting our results.

First, the methods and units of measuring fish intake varied across studies. For example, since the fish consumption categories were not very clear in some studies, we reported only the lowest and highest categories. This may have been an important source of heterogeneity among these studies.

The second limitation is that we only searched the PubMed database. Searching of other databases, such as EMBASE, CANCERLIT and BIOSIS PREVIEWS, may have identified related studies that were not included in our meta-analysis. Because of this limitation, our results should be interpreted cautiously. Future studies including all available databases should test the validity of our conclusions.

The third limitation is that we included only studies that were published in English, thereby potentially excluding several useful studies.

Our fourth limitation is that our searching strategy used the terms case-control studies [MeSH] OR retrospective studies [MeSH] OR cohort studies [MeSH] OR prospective studies [MeSH]. This may have led to the exclusion of useful studies not indexed by any of these terms.

Lastly, our study is limited because of the small sample size of 17 studies. The further sensitivity analysis restriction may therefore have led to loss of statistical significance for pooled relative risks, although the risk estimates changed only slightly. Because of the small sample size, we had limited power to conclusively reject the null hypothesis of no publication bias. Therefore, we set statistical significance for publication bias at P < 0.10. However, publication bias was not detected, either visually or by the Egger's test.

## Conclusions

In summary, from the present meta-analysis we still cannot draw conclusion that fish consumption has preventive effects on gastric cancer. Additional epidemiological studies on the association between diet and cancer are needed to reach more definitive conclusions. These studies should focus on the incidence of gastric cancer relative to different dietary categories, different levels of fish consumption, or different types of fish in the diet.

## Competing interests

The authors declare that they have no competing interests.

## Authors' contributions

SW, JL, XZ and DM conceived and designed the study. XZ and XL did the literature search and identified eligible studies. SW, JL and XZ coded articles and decided on their inclusion. SW, XL and LZ did statistical analyses and interpreted results. All drafts of the reports, including the final version, were written by SW and JL and revised by DM All authors read and approved the final paper.

## Pre-publication history

The pre-publication history for this paper can be accessed here:

http://www.biomedcentral.com/1471-2407/11/26/prepub
